# What makes an academic paper useful for health policy?

**DOI:** 10.1186/s12916-015-0544-8

**Published:** 2015-12-17

**Authors:** Christopher J. M. Whitty

**Affiliations:** London School of Hygiene & Tropical Medicine, Keppel Street, London, UK

**Keywords:** Anthropology, Economics, Policy, Politics, Social science, Synthesis, Systematic reviews, Trials

## Abstract

Evidence-based policy ensures that the best interventions are effectively implemented. Integrating rigorous, relevant science into policy is therefore essential. Barriers include the evidence not being there; lack of demand by policymakers; academics not producing rigorous, relevant papers within the timeframe of the policy cycle. This piece addresses the last problem. Academics underestimate the speed of the policy process, and publish excellent papers after a policy decision rather than good ones before it. To be useful in policy, papers must be at least as rigorous about reporting their methods as for other academic uses. Papers which are as simple as possible (but no simpler) are most likely to be taken up in policy. Most policy questions have many scientific questions, from different disciplines, within them. The accurate synthesis of existing information is the most important single offering by academics to the policy process. Since policymakers are making economic decisions, economic analysis is central, as are the qualitative social sciences. Models should, wherever possible, allow policymakers to vary assumptions. Objective, rigorous, original studies from multiple disciplines relevant to a policy question need to be synthesized before being incorporated into policy.

## Introduction

Health and science policy can mean many things, but in this paper, it is the decisions taken by regional, national or multilateral organisations that aim to have an impact on health, both international and domestic. These may be decisions on resource allocation, legislation or practice guidelines. Policy decisions are invariably weakened when they do not take account of the best current knowledge. Incorporating relevant research findings into policy and practice should therefore be central to the aims of those undertaking practically oriented health research, including in the basic and social sciences. There are a number of reasons policy decisions are not more evidence-based but three predominate. The first is simply that the research has not been conducted; for many important policy decisions it is impossible to be evidence-based because the evidence is currently not there. This is the responsibility and skill-set of academia, although policy-makers can help prioritize key questions. The second is a demand-side problem, with policymakers unwilling or unable to take account of good existing evidence. Those of us in the academic community often blame this demand-side weakness, but at least as much of a barrier is a supply-side problem; the academic community is often weak in producing papers usable in policy even when the evidence is there.

It is this third problem that this discussion piece sets out to explore: how can academics write papers that are more likely to be useful within policy? It should be the easiest to fix if, as academics and scientists, we are serious about trying to improve policy. I was asked to write it having just stopped being Chief Scientific Adviser at the UK Department for International Development (DFID). This role was an interface between science and policy, and I briefly also acted as director of policy. It is therefore one person’s view rather than a consensus statement, with a bias to international development, but the points made are likely to be common ground to most policymakers trying to get policy based on the best available science. What makes a good scientific policy paper, defined as a paper likely to influence and improve policy decisions based on science?

## Discussion

The starting point for any piece of communication, and a scientific paper is a form of communication, is: who is the audience? In one sense, anybody who makes decisions is a policymaker. A guideline writing group or a hospital management committee are. Policymakers discussed in this paper, however, are those having to operate at a larger, generally national or multinational scale. Whilst there are many variations on this, there are some common features which are often forgotten by those who think they are writing for policymakers.

The first is the relatively limited range of things policymakers can do. They can exhort; in some cases they can legislate; most commonly they deploy resources but this means moving resources from one priority to another rather than creating new resource. The second follows from the fact that their main lever is moving constrained resources around and choosing between different ratios of financial allocation; they tend to think like economists or be advised by them, and economics is the training for many policymakers, whether civil servants or politicians. This does not mean good economic analysis is essential to influence policy (although it certainly helps), but good policymakers will always be asking the question ‘what is the opportunity cost of this new initiative?’. Policymakers also therefore can be more numerate than scientists often give them credit for, and have access to well trained statisticians.

Three other elements of the policy-making environment need to be borne in mind. Policy-making is generally extremely fast by academic standards. Many quite complex policy decisions are taken in days, weeks or at most a few months, the time it takes a competent PhD student to begin an introductory chapter. This leaves almost no time for pulling complex evidence together once the policy process is underway, which therefore has to be done in advance. The second is that policies fall on a continuum between purely political decisions, and purely technical decisions. A political decision might, for example, be whether to allocate resources to primary schools or stroke care; science has little to offer in this choice. A purely technical decision is which drug to use for thrombolysis in stroke in which political considerations should usually play no part. Most difficult policy problems lie between these two extremes. Policymakers are assailed on all sides by people with strong opinions pushing their point of view. The evidence-light (or evidence-free) opinions of distinguished scientists are of no more use than any other advocacy group. If scientists take a biased approach to the evidence they quote, or use positional power to give their personal opinion extra weight, they are positively damaging to rational policy-making and may well harm vulnerable people by persuading policymakers to be irrational in the allocation of resources in a way that leads to net loss.

Finally, it is rare that all the evidence needed for a moderately complex policy problem comes from a single discipline, and rarer still that it comes from a single study. As an example, if you consider a seemingly relatively simple question like how best to target antimalarial drugs in Africa, a serious answer will need contributions from several basic sciences, epidemiology, clinical trials, anthropology and economics, and the answer may vary by cultural and epidemiological setting. Despite the multidisciplinary nature of the decision-making process, papers that address only one aspect rigorously are still extremely useful for policy. Improving the evidence base and reducing uncertainty for every link in the assumed chain of causality between a policy decision and its intended outcome is valuable. It does, however, mean that leaping from a single aspect of a problem (most scientific studies) to the proposed solution should be done with due caution, and a degree of humility.

### Principles of what makes a good policy paper (and what does not)

If you compare papers used by policymakers that are genuinely useful for improving the evidence base for policy making with those that are often published as a ‘policy paper’ or in the policy section of journals, the mismatch is often considerable. Many of these ‘policy papers’ are simply footnoted opinion pieces, or even naked advocacy. Others start from the policy decision individual scientists think should be taken rather than from the evidence around the problem that needs to be solved, and suffer from all the problems which occur when intelligent people start with a solution and try and fit the problem to it. Others offer policy advice which would work fine if we consider only the science relevant to their discipline and ignore all the inconvenient complexities brought in by other relevant sciences, including the social sciences. This is not a new problem, but what Sir Francis Bacon, an early scientist-policymaker called the *‘idols of the cave’*, the tendency to give disproportionate weight to your own intellectual tradition [1]. Finally, some scientists appear to believe that a paper for policymakers, which might affect vulnerable lives, requires less intellectual and methodological rigour than one aimed at their academic peers; this is incorrect.

Fortunately, there are a wide range of types of paper which have considerable use to inform policy and which can be very influential. They have some things in common.

1. They state explicitly the policy problem or aspect of a policy problem the paper addresses. This makes them easy to identify. A policy problem is not usually the same as a scientific problem, and may have several scientific problems incorporated within it.
2. They are explicit about methodologies, limitations and weaknesses. This may sound obvious to writers from some scientific traditions but, for example, in many social sciences, very limited methods may be outlined in reputable journals [2]. The technical part of any policy team should be trying to assess the strength of each bit of evidence used, whether via formal grading system as used in medical guidelines or more informally [3]. Doing this without methodologies laid out is nearly impossible.
3. The authors have made a serious attempt to minimise their own biases in both methodology and interpretation. Scientists can be advocates, or they can provide the best possible balanced assessment of the evidence but they cannot do both simultaneously. It has to be clear to policymakers which horse they are riding. Papers seen as advocacy are likely to be discounted.
4. Since the policy process tends to be very fast, papers must be timely. An 80 % right paper before a policy decision is made it is worth ten 95 % right papers afterwards, provided the methodological limitations imposed by doing it fast are made clear. The use of fast-tracking by journals seems more logical for papers because they are time-limited in their impact than because they are deemed important.
5. Remembering that the audience may be intelligent laypeople authors should apply the aphorism attributed to Einstein; be as simple as possible (but no simpler) in methods and language. Authors, referees and editors can transform papers making a simple point using methods easily understood by policymakers into Baroque complexity understood only by modelers or statisticians by insisting on using techniques irrelevant to the key policy message which only add a spurious level of precision. Some papers (especially but not exclusively in the social science journals) use unnecessarily complex language or redundant jargon. Sensible policymakers prefer a paper they understand, including its flaws, to one they do not, however sophisticated and apparently precise it looks. It is possible to be simple whilst being rigorous.
6. Describing the problem that needs resolving is only useful until the description is clear, and policymakers understand there needs to be action. Then the policy question needs to be asked: what is the evidence about the available options for things we can do to resolve the problem? This should be obvious, but it is surprising how many scientists continue to describe a problem in greater and greater detail for years after policymakers have clocked it, without going the next step of designing and testing interventions.
7. Don’t feel the need to spell out policy implications. This may sound counter-intuitive, but many good scientific papers are let down by simplistic, grandiose or silly policy implications sections. Policymaking is a professional skill; most scientists have no experience of it and it shows. In DFID, we stopped asking people undertaking commissioned systematic reviews to write a ‘policy implications’ summary of their review. This was because the understanding of the real policy questions were usually poor even when the review was itself very well done and therefore undermined the paper. Worse, trying to work up to a policy position can unconsciously bias scientists towards trying to get a neat policy narrative from a complex picture, or downplay inconvenient facts. Therefore, in general, the data collection and analysis process and the policy process are best kept separate. If you feel it is useful to give your policy analysis based on your data be modest: few papers underestimate their policy importance, many substantially overestimate it and many do not provide the social context.

## Types of paper most commonly useful in policy

### Synthesis

Way ahead of any other academic contribution to policy-making is rigorous and unbiased synthesis of current knowledge. It is an unfortunate side-effect of the incentives built into academic life, including the UK Research Excellence Framework (REF), that there is a tendency to try and claim an individual study as the basis for a policy decision. In reality, policy should always be based on the whole sweep of current scientific knowledge, usually from multiple disciplines. Some scientists seem to assume a two-stage process where the individual research is conducted, and then policies made on the basis of that. The reality should be a three-stage process: that original research is conducted, then research from multiple instances and disciplines is synthesised, and then policies made on the basis of all the available synthesized evidence. Unfortunately, despite accurate synthesis being by far the most useful academic skill for policy-making, it tends to have low prestige in the academic community, a form of snobbery which is very unhelpful if we believe our own rhetoric that science can and should change the world.

Health-related sciences have contributed a major tool for synthesis which is systematic reviews. These are now being increasingly used in other disciplines for policy [4]. The general principles of systematic reviews of a trawl for evidence relevant to the policy question to avoid positive publication bias, followed by a quality filter are widely applicable. Systematic reviews in the narrower and sometimes theological definition of Cochrane or Campbell reviews often discard anything which is not a randomised trial. Unfortunately, this means that a large proportion of the papers most relevant to policy, but which do not have experimental quantitative data, despite being in their own way rigorous, are also discarded. Systematic reviews are therefore often necessary, but certainly not sufficient. Many important policy questions (as opposed to clinical questions) are not amenable to trials. It should be acknowledged also that many papers labeled as systematic reviews are methodologically weak or poorly conceived; a bad systematic review should have no place in the GRADE system, but there are a lot out there. In particular there is a tendency to jump from the systematic review to an inappropriate meta-analysis. If the academic community as a whole could do one thing to improve the pathway from research to policy, it would be to improve the status, quality and availability of good synthesis. For example some current initiatives by the UK Economic and Social Research Council (ESRC) into new methods for synthesizing social science are an encouraging development [5].

### Papers which challenge current thinking with data

Most well-trained policymakers find papers which challenge or modify their assumptions extremely useful. It is far better (and less embarrassing) to get a challenge before a decision is made, than to realise you have misunderstood it afterwards. A challenge, clarification or gloss on current thinking has weight if it is based on solid data neutrally presented, but not when based only on opinion. A data-based paper which makes a single policy point well backed up and with all the limitations laid out is frequently extremely influential in policy decisions. The more points that are loaded onto a paper the less easy it is for the policy process to digest it so making one point well backed by rigorous analysis will generally have greater impact than a complex analysis of multiple policy points.

### Models and economic models

Models are frequently used to predict the possible impacts of a change in policy, or its cost-benefit implications. Models unfortunately tend to induce extreme reactions in most people, including policymakers, who are not modelers. Either they tend to believe them completely, or ignore them completely. Clearly the right place is between the two. The tendency of some modelers to present them as scientific predictions of the future rather than models does not help. Models are widely used in government, and some models arguably have too much influence. They are generally most useful when they identify impacts of policy decisions which are not predictable by commonsense; the key is usually not that they are ‘right’, but that they provide an unpredicted insight. A good recent example was the model which predicted the upswing of Ebola in West Africa and helped spur (belated) international action [6]. The model was not ‘right’ in that it did not predict accurately which countries would be most heavily affected, but it conveyed very clearly the implications of an epidemic which would compound up over time.

Authors of modelling papers can do things to make their work substantially more useful for policy. The best is to provide an interactive interface, where if the policymaker does not agree with the starting assumptions of the model, they can change them. This is particularly important for the key variables, but also anything which has a big impact on the model outcomes in sensitivity analysis. A nice example from outside the health field is this model for making carbon reduction decisions, which was written by a combined science and policy team [7]. This should be very easy to do for any model. One of the major weaknesses of many models is that they make assumptions based on expert opinion where there are no reliable data, and expert opinion can vary widely [8]. Providing the reader with the opportunity to put in their own assumptions makes intellectual as well as communication sense. Keeping the model simple is also important. There is likely to be an inverse relationship between the complexity of the model (making its workings incomprehensible to most readers) and its uptake by policymakers who have a reasonable understanding of modeling but do not trust black box models.

Economic models, in particular from microeconomics (of which most health economics is a subset) are in a different category because so many policymakers are trained in economics. They generally have fewer problem in understanding economic models including their strengths and weaknesses.

### Papers from the social sciences

The great majority of policy initiatives, including health, involve some behavioral aspects, and most involve behavior change. Many policy decisions do not turn out the way they were intended because people do not behave in the way policymakers and scientists thought they would, or should. There is a wide open goal for timely, relevant, rigorous and readable qualitative and quantitative social science addressing practical questions in policymaking. The supply of such research is thin compared to the demand, and methods for synthesizing it in a rigorous way are not as well developed or universally agreed as in clinical medicine [9]. A surprising number of people who conduct trials do not, alongside of this, conduct the social science research which would provide the context to make them translatable into policy. A lot of excellent (in the sense it is academically well done) social research is conducted, but the amount that is usable for policy is a fraction of this. Some ostensibly policy-relevant social research is methodologically weak. As an academic community, we need to address this seriously; with the exception of economics the social sciences punch well below their weight in policy, and in my experience this is more a supply-side than a demand-side problem.

### Trials

Few methodologies are clearer, and easier to communicate, than a trial. Like clinicians, policymakers generally like them because they give a quantitative dichotomous answer (it worked or it did not, and by how much) to a question about which intervention works best. A good trial is difficult to argue with, and even a methodologically weak trial can get a lot of traction. They are relatively easy to turn into an economic analysis on cost-benefit or cost-effectiveness, although despite this being a key policy consideration, surprisingly few triallists do this except as an afterthought. Not all policy questions suit a trial design, but many do. From a policy perspective, the main problem with many trials is that they are a form of efficacy trial (ideal world) rather than effectiveness or pragmatic trial (what would happen under realistic circumstances). Anybody who understands trials knows that there is often a major gap between efficacy and effectiveness [10]. Making policy on the basis of efficacy trials may be better than nothing, but it would be much better also to have a (costed) effectiveness trial, and preferably more than one backed up by economic and social analysis.

## Conclusions

Getting relevant science and research into policy is essential. There are several barriers, but the easiest to reduce is making papers more relevant and accessible to policymakers. Opinion pieces backed up by footnotes are generally unusable for policy. Objective, rigorous, simply written original papers from multiple disciplines with data can be very helpful. These then need to be well synthesized.
